# Multilabel Image Classification Based Fresh Concrete Mix Proportion Monitoring Using Improved Convolutional Neural Network

**DOI:** 10.3390/s20164638

**Published:** 2020-08-18

**Authors:** Han Yang, Shuang-Jian Jiao, Feng-De Yin

**Affiliations:** Department of Civil Engineering, College of Engineering, Ocean University of China, Qingdao 266100, China; jiaoaiouc@163.com (S.-J.J.); fdyin_ouc@163.com (F.-D.Y.)

**Keywords:** multilabel image classification, convolutional neural network, mix proportion, real-time monitoring, integrated intelligent sensing system, intelligent manufacturing and construction

## Abstract

Proper and accurate mix proportion is deemed to be crucial for the concrete in service to implement its structural functions in a specific environment and structure. Neither existing testing methods nor previous studies have, to date, addressed the problem of real-time and full-scale monitoring of fresh concrete mix proportion during manufacturing. Green manufacturing and safety construction are hindered by such defects. In this study, a state-of-the-art method based on improved convolutional neural network multilabel image classification is presented for mix proportion monitoring. Elaborately planned, uniformly distributed, widely covered and high-quality images of concrete mixtures were collected as dataset during experiments. Four convolutional neural networks were improved or fine-tuned based on two solutions for multilabel image classification problems, since original networks are tailored for single-label multiclassification tasks, but mix proportions are determined by multiple parameters. Various metrices for effectiveness evaluation of training and testing all indicated that four improved network models showed outstanding learning and generalization ability during training and testing. The best-performing one was embedded into executable application and equipped with hardware facilities to establish fresh concrete mix proportion monitoring system. Such system was deployed to terminals and united with mechanical and weighing sensors to establish integrated intelligent sensing system. Fresh concrete mix proportion real-time and full-scale monitoring and inaccurate mix proportion sensing and warning could be achieved simply by taking pictures and feeding pictures into such sensing system instead of conducting experiments in laboratory after specimen retention.

## 1. Introduction

Mix proportion refers to the components and their proportions of concrete. Both theoretical guidance and engineering practice adopt property-based mix proportion design method currently, leading to a fact that in a specific environment and structure, properties and structural functions of concrete in service are enormously affected by its mix proportion. A neglectable error of mix proportion could result in great difference of concrete service behavior [1].

Modern concrete has been, to date, the most widely used building material in all kinds of construction engineering since its invention because of its irreplaceable and incomparable advantages. Due to the rapid pace of urbanization and the explosive growth of global infrastructure market, the consumption of cement-based concrete is considerable. It is estimated that global concrete consumption exceeds 3.3 × 10^10^ t annually [1]. On the other hand, China national standards [2,3] stipulate that during construction process, on-site sampling test and specimen retention shall not be less than once every 200 m^3^ when concrete mixture is continuously poured over 1000 m^3^ for the same batch with the same mix proportion, and various items are involved in on-site sampling test such as mixture test, compressive strength test and durability test, green manufacturing and resource saving are hindered by such tests.

Moreover, during manufacturing, mechanical and weighing sensors are commonly applied to ensure accurate quantities of raw materials entering mixing process at present. The failure of mechanical and weighing sensors is one of the most momentous reasons leading to inaccurate mix proportions of concrete product. Simultaneously, existing testing methods represented by experiments after a 28-day standard curing are all posterior approaches, structural deficiency caused by inaccurate mix proportion usually could not be discovered until concrete is hardened and strength is taken shape. The joint effect of the delayed testing in production process and the property-based mix proportion design method prominently raises the risk of engineering accident on construction site. Additionally, existing testing method is a low-sampling-rate inspection method due to its essential characteristic of consuming products themselves and human-conducted on-site inspections need appreciable number of well-educated professional technicians. Therefore, we contend that concrete quality monitoring at early ages, even fresh concrete is highly desired. There is a pressing need to apply intelligent method as an effective complement to mechanical and weighing sensors and existing test methods, to achieve timely, full-scale and nonconsumption monitoring and sensing of fresh concrete mix proportion during manufacturing.

This paper intends to present a novel deep-learning-based method for real-time and full-scale monitoring and sensing of fresh concrete mix proportion. The present study is built on a powerful tool in deep learning community, that is, convolutional neural network (CNN). The novelty of this method lies in introducing a state-of-the-art technology to conventional and well-developed construction material field, which could achieve mix proportion monitoring during manufacturing simply by taking pictures rather than conducting experiment in laboratory and could sensing inaccurate mix proportions by processing images directly without transforming and coping with numeric data. We aim to (1) train CNN with desired learning and generalization ability to identify mix proportion and (2) apply the well-trained CNN to engineering practice and united it with existing mechanical and weighing sensors to establish integrated intelligent sensing system. As a typical data-driven, learning-based approach, this will be done by solving the following research questions: (1) how to design and setup experiment and collect data since an elaborately planned, uniformly distributed, widely covered and high-quality dataset is crucial for the success of a typical CNN model and (2) how to improve and fine-tune CNN to simultaneously identify multiple mix proportion parameters since typical CNNs are tailored for single-label multiclassification tasks.

According to abovementioned framework, in this paper, we discuss related works and current state of research in Section 2, elaborate on data collection process, the research methodology and the establishing and improving of CNN models to implement multilabel image classification in Section 3 and Section 4, respectively, the selection of the best-performing CNN and its application in engineering practice are elaborated in Section 5.

## 2. Related Works

Much effort has been devoted to detecting concrete properties or identifying mix proportion by previous studies.

In the past few decades, prediction of concrete mechanical properties seemed poised for an explosive adoption of machine learning algorithms. Many studies [4,5,6,7,8,9,10] dedicated to adopting various models, including artificial neural network (ANN), genetic algorithm, support vector machine (SVM), and so forth, to predict properties, especially mechanical properties represented by compressive strength of concrete. Numeric data such as mix proportions and curing conditions were used by these studies as inputs of different models.

With respect to the application of image processing, computer vision and deep learning methods, Han et al. [11] developed two-dimensional image analysis method based on concrete cross-section image to evaluate coarse aggregate characteristic and distribution. Başyiğit et al. [12] applied image processing technique to assess compressive strength. Dogan et al. [13] used artificial neural networks and image processing together to identify mechanical properties. Wang et al. [14] introduced digital image processing method to evaluate binary images and predict flowability using cross sectional of self-consolidating concrete. Hyun Kyu Shin et al. [15] adopted deep convolutional neural network to develop a digital vision-based evaluating model, so as to predict concrete compressive strength with surface images. All of these studies were tailored for images of hardened concrete, which would encounter huge challenges when dealing with the situations that require timely detection.

As for early estimating properties of concrete, some studies and theoretical guidance [16] also concentrate a significant amount of attention. To date, relatively mature early estimating approaches include accelerated curing method, microwave heating method, mortar accelerated setting with pressure curing method, and so forth. These approaches are usually complicated and establishing empirical equation through experiments is always required. It takes at least 1.5 h to carry out experiments for these methods, which are not poised for real-time monitoring.

It is worth noting that aforementioned studies all devoted their efforts on predicting or identifying concrete properties. In practice, however, the focus is not the properties, but mix proportions, since most service behaviors always have low level of bias with the properties derived from mix proportion, that is, to a large extent, properties are determined when mix proportion is fixed because of the property-based mix proportion design method. However, the mix proportions in practice are not always keep in line with their calculated values due to the failure of production equipment. Therefore, monitoring mix proportion is deemed to be crucial for the success of estimating or evaluation concrete service behaviors.

With respect to mix proportion monitoring or identification, to name a few, Jung et al. [17] proposed a new fingerprinting method and adopted empirical equation to determine mix proportion by acid neutralization capacity (ANC) of concrete. Chung et al. [18] presented mathematical models for monitoring mix proportions based on dielectric constant measurement. Philippidis et al. [19] adopted acousto-ultrasonics method and time and frequency domain schemes for the evaluation of water-to-cement ratio. Chung et al. [20] applied microwave near-field noninvasive testing technique to determine initial water-to-cement ratio. Bois et al. [21] presented a near-field microwave inspection technique for early determination of water-to-cement ratio of Portland cement-based materials. The so-called “early ages” in such studies also require two days at the earliest. In line with the studies aforementioned, these studies also could not address the limitations of timeliness and product consumption.

When it comes to applying deep learning approaches to mix proportion real-time monitoring of fresh concrete, only our previous work [22] that introduced pre-trained CNNs to detect water-to-binder ratio is found, and no further studies are discovered. We therefore contend that using fresh concrete images for mix proportion real-time monitoring and sensing with deep learning methods has yet to be solved.

## 3. Data Collection

### 3.1. Mix Proportion Design

The present study only focuses on ordinary concrete that consists of water, cement as cementitious material, sand as fine aggregate and stone as coarse aggregate (i.e., does not contain additional mineral admixtures, chemical admixtures and other specific components such as recycled aggregate). Accounting for feasibility and mix proportion design in engineering and manufacturing practice, unit water dosage was kept at constant value of 200 kg/m^3^, a fixed mix proportion could be then specified by unit dosages of other three components. Instead of directly used, proportions or quantities of these four components are usually converted into two dimensionless quantities for discussion, namely, water-to-binder-ratio (w/b) and sand-to-aggregate-ratio (s/a).Meanwhile, given the fact that the density of ordinary concrete is 2350–2450 kg/m^3^ [23], unit dosages of three components could be then uniquely determined by three independent numeric relationships, that is, w/b, s/a and the density. We therefore contend that one certain specific mix proportion could be determined by w/b and s/a.

In engineering practice, w/b and s/a always exert enormous influences on concrete service behaviors. Moreover, the nominal maximum particle size of coarse aggregate (NMSCA) also has considerable influence on fluidity and mix proportion design. Therefore, w/b, s/a and NMSCA are considered as three variables for mix proportion design, also three labels of concrete mixture images, classification target objects of CNN models and target objects of mix proportion monitoring.

According to relevant materials [23,24] and engineering practice, maximum and minimum values of three labels were determined. Compressive strength is significantly impacted by w/b and minimum w/b to ensure complete hydration is about 0.25–0.3, while large w/b may cause mixtures bleeding, reasonable w/b is about 0.3–0.7; s/a has enormous implications for fluidity, both too large and small s/a would lead to the declining of fluidity, recommended range of s/a is about 25%–45%; large coarse aggregate particles may increase the number of weak links in harden concrete and further affect mechanical properties, recommended NMSCA is no larger than 31.5 mm.

Within the maximum and minimum values, w/b and s/a were divided into five classes, and NMSCA was divided into three classes, as shown in Table 1.

A total of 75 mix proportions could be obtained by various combinations of w/b, s/a and NMSCA. A few extreme combinations that could not be adopted in engineering practice were excluded and 67 experiments were conducted. Such exclusions were considered form the perspective of compressive strength and fluidity, concrete mixtures with small w/b and large s/a always have poor fluidity, and the ones with large w/b usually have small compressive strength since the latter and the reciprocal of the former show a close linear relationship when the type of cement is determined. Eight excluded mix proportions are listed in Table 2.

### 3.2. Materials

Ordinary Portland cement produced in Tangshan, Hebei province was selected in this study. Its basic physical and mechanical properties are shown in Table 3. After inspection, its basic physical and mechanical properties and the content of chemical components conform to China national standard [25].

River sand with moderate fineness produced in Qinhuangdao, Hebei province was selected as fine aggregate. Its major physical properties conform to China national standard [26], as listed in Table 4.

Crushed stone was chosen as coarse aggregate. Table 4 indicates its major physical properties. When NMSCA = 20 mm or 31.5 mm, referring to relevant material [27], continuous size grading was adopted to improve concrete service behaviors, as listed in Table 5.

### 3.3. Experiment Setup and Image Collection

All the experiments in this study were conducted in Beijing from May to July in 2019. HJW60 concrete mixer, produced in Wuxi, was used for concrete mixing. Well-mixed concrete mixtures were poured into the metal tray with the size of 800 mm × 800 mm.

In order to ensure the evenness of collected images and reduce the influence of light, indoor light sources were controlled, and a 1-m-high metal plate was fixed on each side of the tray to block uneven light. Hand-held image acquisition device took pictures at a fixed height of 400 mm from the bottom of the tray to reduce accidental error. Image collection was completed within 120 s after pouring to avoid slump loss, maintain freshness and keep in line with manufacturing practice.

The same number of image sets obtained from 67 experiments include a total of 8340 images, containing 152 images for the set with the maximum number and 82 with the minimum.

### 3.4. Image Preprocessing, Data Augmentation and Dataset Segmentation

Few blurred images and images with too much noise were deleted. In each set, one image was randomly selected as testing image to set up a testing set containing 67 images. Data augmentation was carried out for the rest images and the number of images in each set was expanded to 200 in order to improve generalization ability of CNN models and prevent networks from overfitting. Given the fact that existing ones could cover all poured mixtures in the tray, we did not add more images to avoid networks memorizing exact details, since more images lead to more detail repetitions. Approaches for data augmentation include rotation, horizontal shift, vertical shift, shear, zoom and horizontal flip.

200 images in each set were randomly divided into training and validation set after shuffling, of which training set was made up for 75% and validation set for 25%. Therefore, there are 10,050 images in training set and 3350 in validation set.

## 4. Methodology

### 4.1. Deep Learning Theory and CNN

Deep learning methods are representation-learning methods. Multiple levels of representation were obtained by composing nonlinear, but simple modules that each transform the representation at one level into a more abstract and complex level [28]. Instead of extracting features manually, deep learning methods allow a machine to be fed with raw data, and a number of filters then extract the topological features hidden in input data and needed representations were automatically discovered, this is also the essence of deep learning methods [28,29].

CNN is a kind of typical, outstanding and most widely adopted basic structure of deep learning theory. LeCun et al. [30] published a paper establishing basic framework of CNN and later improved it using gradient-based optimization for document recognition [31,32]. The architecture of a typical CNN is structured as a series of blocks. The first few blocks are composed of combinations of convolutional layers and pooling layers, and the last block usually consists of fully connected layers and a classification model [28,29,33]. Four key insights behind CNN that take advantage of the properties of natural signals are shared weights, local connections, pooling and the use of many layers [28].

There are also many other approaches with successful applications in deep learning community. These neural networks are tailored for a variety of different tasks, to name a few, recurrent neural networks (RNN) [34] and long short-term memory (LSTM) [35,36] are suitable for modeling sequential data and sequence recognition and prediction, region-based convolutional neural network (R-CNN) [37] as well as its variants and you only look once (YOLO) [38] are capable of tackling object detection problems, SegNet [39] is tailored for semantic segmentation tasks. Such models have more complex structures and modules, such as memory blocks in LSTM, to cope with more complicated problems [40,41]. Complex structures correspond to considerable computing resources consumptions. Classification is one of the most basic tasks for deep learning, so neural networks for classification tasks usually have simple, but effective structures, and computational costs could also be reduced.

Widely used CNNs for our image classification tasks include AlexNet [42], VGGNet [43], GoogLeNet [44], ResNet [45], and so forth.

### 4.2. Multilabel Image Classification

Real-world objects always have multiple semantic meanings simultaneously. The paradigm of multilabel learning emerges to deal with the defect that explicit expression of such multiplicity is hindered by the simplified assumption of traditional supervised learning that each real-world object is represented by a single instance and associated with a single label [46]. Compared with single label classification, more information is asked for multilabel classification, but it is more similar to human cognition.

To cope with the challenge of exponential-sized output space, it is crucial to effective exploit label correlations information. Existing strategies could be roughly characterized into three families based on the order of correlations. First-order strategy tackles learning tasks in a label-by-label style and thus ignoring coexistence of the other labels. Second-order strategy considers pairwise relations between labels. High-order strategy considers high-order relations among labels [46].

In machine learning community, solutions to multilabel image classification are usually considered from two perspectives. Problem transformation methods include label-based transformation and instance-based transformation, which fit data to algorithm and transform multilabel tasks into other well-established learning scenarios, especially single-label classification. Representative algorithms include binary relevance [47], classifier chains [48], calibrated label ranking [49], random *k*-labelsets [50], and so forth. Algorithm adaptation methods fit algorithm to data and adapt successful learning techniques to deal with multilabel data directly. Typical algorithms include ML-kNN [51], ML-DT [52], rank-SVM [53], CML [54], and so forth.

Particularly, with respect to deep learning, the application of CNN for multilabel image classification is rapid widened because of the prominent merits aforementioned. According to the key philosophy of problem transformation methods, CNN could be directly used for multilabel classification tasks, such methods were named as “multilabel CNN” [46,55]. CNN could also be improved or combined with other algorithms, outstanding methods include HCP [56], CNN–RNN [57], RLSD [58]. Such improved CNNs usually apply a feature selection attention strategy called attention mechanism. In addition, brand new algorithms represented by GCN [59] are developing recent years.

### 4.3. Establishing and Improving of Network Models

In this study, according to aforementioned solutions for multilabel image classification tasks, four CNN models were established for training and comparative study, among which the best-performing one would be selected and put into manufacturing and engineering application for mix proportion monitoring.

#### 4.3.1. CNN Models Based on Problem Transformation

Multilabel classification task for w/b, s/a and NMSCA was transformed into multiclassification task for mix proportion “instance”. Specifically, corresponding to 67 experiments, different classes of three labels were combined to obtain 67 sets of instances of mix proportion, and concrete mixture images were classified into 67 classes at the level of mix proportion instances. Two kinds of original multiclassification-based CNN structures were applied to realize the classification of mix proportion instances.

AlexNet structure was adopted as the first basic CNN structure since it is a breakthrough in the development of CNNs that has simple, but efficient network configuration. The advantage of AlexNet lies in its two independent GPUs for simultaneous training, and actually its layers and filters are structured in groups. Furthermore, for the second, fourth and fifth convolutional layers, the convolution kernels deployed on a specific GPU is only connected with the ones on the same GPU in the previous layer. Evidently, such structures are not applicable to limited hardware facilities. In addition, it is worth noting that input images were classified into 1000 classes by original AlexNet, such number is significantly larger than that of mix proportion instances. Therefore, AlexNet structure was fine-tuned as follows to adapt to mix proportion classification task: (1) Structure about double parallel-working GPUs was ignored, convolution kernels separately deployed on two GPUs were merged and local response normalization (LRN) operation was replaced by batch normalization (BN) and (2) the last fully connected layer and output layer were reset to classify concrete mixture images into 67 classes. For further discussion, this fine-tuned AlexNet-structure-based CNN was named as Net-I.

AlexNet has a considerable number of parameters that exceed 61 million since ImageNet dataset [60] was used for its training, of which the number of classes and images are significantly larger than that of concrete mixture dataset. Given that training a network which has too many parameters with a relatively small dataset may lead to overfitting and further resulting in the declining of generalization ability, a simpler CNN with fewer parameters was established referring to AlexNet structure. Input size of images, network depth, the number of layers, the number and size of filters were comprehensively considered. After a trial-and-error process, detailed structure of the simple CNN was determined as illustrated in Table 6. This self-established simple network was named as Net-II for further discussion.

#### 4.3.2. CNN Models Based on Algorithm Adaption

Although above-discussed method, adapting dataset to original multiclassification-based CNN, is simple and convenient to perform, the prominent defect lies in the sample loss. To be specific, for images of a given class in the corresponding label, such as w/b = 0.3, rather than being gathered up to enable networks to learn the features of concrete mixture images for such class, they were distributed to 10 sets of images with different mix proportions. Moreover, network models could only be evaluated at the level of mix proportion instance, and it could not be estimated that how the networks performed with regard to each of the three labels.

To tackle such defects, improvement of original CNN tailored for multiclassification tasks is desirable. First, for one sample, confidence scores of different classes outputted by Softmax activation function adopted in original CNN are correlated with each other, and all returned values are always summed up to 1. However, for our multilabel classification task, three labels: w/b, s/a and NMSCA are not mutually exclusive. Therefore, Softmax function was replaced by Sigmoid function as the activation of output layer. For the confidence score $P\left(y^{\left(i\right)}=n|x^{\left(i\right)};w\right)$ of the *j*–th class being proper label of the *i*–th example, Sigmoid and Softmax function are defined as Equations (1) and (2), respectively:

(1) $$P_{sigmoid}\left(y^{\left(i\right)}=n|x^{\left(i\right)};w\right)=\frac{1}{1+\left[\begin{array}{c} e^{-w_{1}^{T}x^{\left(i\right)}}\\ e^{-w_{2}^{T}x^{\left(i\right)}}\\ \vdots \\ e^{-w_{n}^{T}x^{\left(i\right)}} \end{array}\right]},$$

(2) $$P_{softmax}\left(y^{\left(i\right)}=n|x^{\left(i\right)};w\right)=\frac{1}{\sum_{j\;=\;1}^{n}e^{w_{j}^{T}x^{\left(i\right)}}}\left[\begin{array}{c} e^{w_{1}^{T}x^{\left(i\right)}}\\ e^{w_{2}^{T}x^{\left(i\right)}}\\ \vdots \\ e^{w_{n}^{T}x^{\left(i\right)}} \end{array}\right],$$

for *i* = 1, 2, …, *m*. Where, *m* is the number of examples at current batch, *n* represents the number of classes, w denotes the weights, $w_{n}^{T}x^{\left(i\right)}$ is the output of the previous layer.

Subsequently, categorical cross entropy was replaced with binary cross entropy as loss function to consider each output label as an independent Bernoulli distribution. The loss function of binary cross entropy and categorical cross entropy are defined as Equations (3) and (4), respectively:

(3) $$\begin{array}{cc} L_{binary}= & -\frac{1}{m}\sum_{i=1}^{m}\sum_{j=1}^{n}1\left\{y^{\left(i\right)}=j\right\}\log\left(\frac{1}{1+e^{-w_{j}^{T}x^{\left(i\right)}}}\right)\\ & \;+\left(1-1\left\{y^{\left(i\right)}=j\right\}\right)\log\left(1-\frac{1}{1+e^{-w_{j}^{T}x^{\left(i\right)}}}\right), \end{array}$$

(4) $$L_{categorical}=-\frac{1}{m}\overset{m}{\underset{i=1}{\sum }}\overset{n}{\underset{j=1}{\sum }}1\left\{y^{\left(i\right)}=j\right\}\log\left(\frac{e^{w_{j}^{T}x^{\left(i\right)}}}{\sum_{k=1}^{n}e^{w_{k}^{T}x^{\left(i\right)}}}\right),$$

where, the new index *k* is introduced to indicate that $\sum_{k=1}^{n}e^{w_{k}^{T}x^{\left(i\right)}}$ is independent of $\sum_{j=1}^{n}1\left\{\cdot \right\}$. The term $1\left\{y^{\left(i\right)}=j\right\}$ is a logical expression that returns ones if a predicted class of the *i*–th example is true for *j*–th class and returns zeros otherwise.

Essentially, above two improvements transform the multilabel classification problem to binary-classification task for each example, that is, CNN models would successively determine whether each of the total 13 classes is or is not a proper label of a given image. Such transformation is realized by adapting CNN models to datasets, so these improved CNN models were still recognized as algorithm-adaption-based models.

It is worth noting that correlations of labels are not considered by above improvements. Specifically, three classes with the highest confidence scores will be outputted by CNN models, but could not be guaranteed that they come from three different mix proportion labels. To cope with such shortcoming, an additional module was added after the output layer to enable network models to select the class with the highest confidence score separately from w/b, s/a and NMSCA as the final outputs.

Corresponding to Net-I; and Net-II, both AlexNet and self-established structure were improved for multilabel image classification. Improved AlexNet and self-established structure were named as Net-III and Net-IV, respectively for further discussion.

Moreover, deep learning tasks usually encounter huge challenges when tuning training hyperparameters. Manual trail-and-error processes may consume considerable time. Therefore, Bayesian optimization [61,62] was adopted to search optimal hyperparameters in parameter space. Bayesian optimization was applied to optimize initial learning rate, batch size and epoch. Approximate search ranges of three hyperparameters were determined by preliminary training in advance. In order to accelerate the convergence of CNN models, instead of setting continuous closed intervals, several discontinuous values were specified within their approximate ranges to form three sets for searching, as manifested in Table 7. The rest major training options were fixed as listed in Table 8.

**Figure 1:**
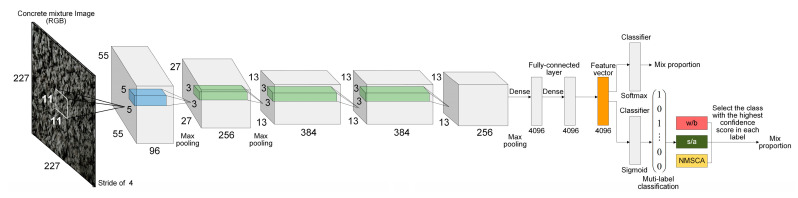
Structure of Net-I (above) and Net-III (below).

**Figure 2:**
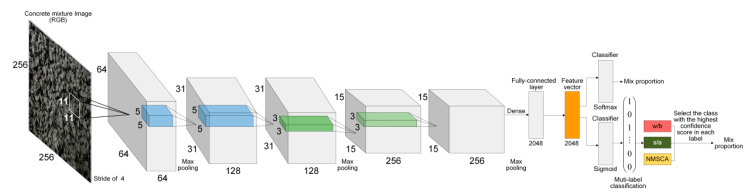
Structure of Net-II (above) and Net-IV (below).

To summarize, adjustments or improvements of four CNN models are summarized in Table 9. Four established CNN models are visualized by Figure 1 and Figure 2.

## 5. Results

All programs in this study were performed with MatlabR2020a and Python3.8, on a desktop computer equipped with 2.6 GHz Intel i7-4720 CPU, 16 GB RAM, x-64-based processor and NVIDIA GeForce GTX960M GPU.

### 5.1. Optimal Hyperparameters Selected by Bayesian Optimization

Bayesian optimization was performed by minimizing classification error of validation set during training, that is, the best-performing CNN model was selected based on validation accuracy. Optimal training hyperparameters selected by Bayesian optimization are illustrated in Table 10.

### 5.2. Metrics and Evaluations of Training and Validation Set

Training with the optimal hyperparameters, training and validation accuracies achieved by four CNN models are listed in Table 11.

Evidently, accuracies on both training and validation set is higher than 99% for all the four CNN models, validation accuracies of Net-III and Net-IV even reached 100%. It is worth noting that both training and validation accuracies of two improved CNN models based on algorithm adaption, Net-III and Net-IV, are higher than that of Net-I and Net-II. Moreover, Net-II and Net-IV—which applied self-established network structures—higher accuracies compared with Net-I and Net-III.

**Figure 3:**
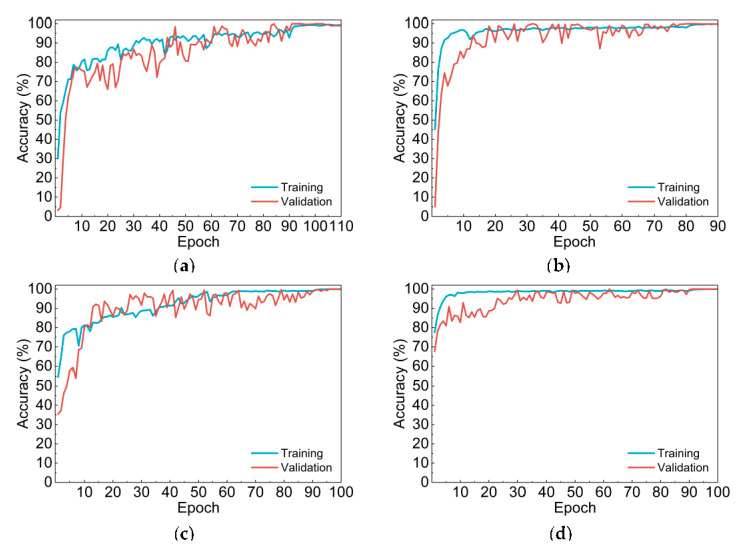
Accuracy curves of four convolutional neural network (CNN) models. (**a**) Net-I; (**b**) Net-II; (**c**) Net-III; (**d**) Net-IV.

Furthermore, refer to accuracy curves shown in Figure 3, compared with multiclassification-based Net-I and Net-II, Net-III and Net-IV had higher initial training and validation accuracies. Net-I and Net-III converged slower and their curves have more significant fluctuations. Meanwhile, time elapsed during training of two CNNs with self-established structure is much shorter than that of Net-I and Net-III, since the number of parameters of self-established CNNs is only 27 million, less than half of AlexNet structure.

**Figure 4:**
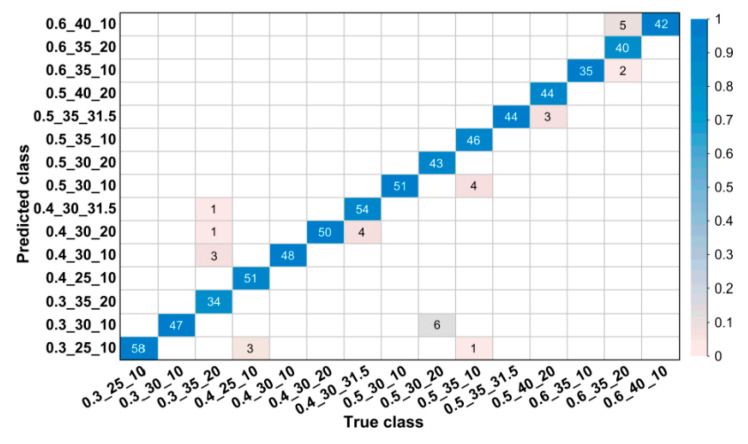
Confusion matrix for validation set of Net-I.

**Figure 5:**
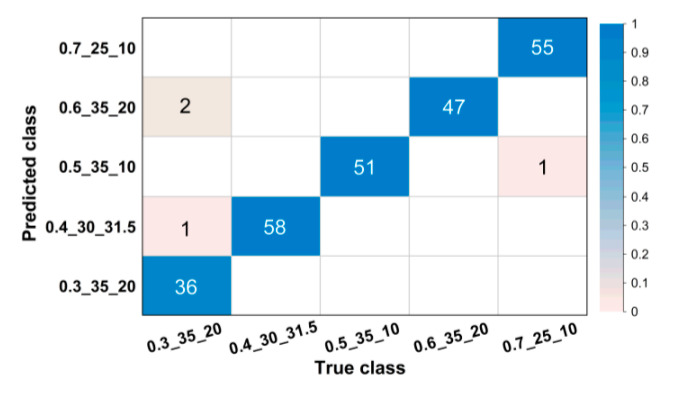
Confusion matrix for validation set of Net-II.

For images in the validation set, confusion matrices were drawn to visually illustrate classification results. Figure 4 and Figure 5 show partial confusion matrices of Net-I and Net-II, it is unnecessary to draw confusion matrices for Net-III and Net-IV because they are not essentially multiclassification models and their validation accuracies reached 100% (name of classes are shown in the form of w/b_s/a_NMSCA, for example, 0.3_25_10 could be interpreted as w/b = 0.3, s/a = 25%, NMSCA = 10 mm, the same hereinafter). Two confusion matrices only show corresponding classes of misclassified images, classes in which all images are correctly classified are not shown here. The element $a_{ij}$ in each cell of the matrix could be interpreted as the number of images classified to the *i*-th class but belong to the *j*-th class. The color of each cell represents its proportion in the sum of all elements in corresponding column, that is, its proportion in the total number of images of corresponding ground truth label and the proportion of correct or incorrect classification. As shown in Figure 4 and Figure 5, most images are in the diagonal of matrices which means correct classification. In the total of 3350 validation images, Net-I misclassified 33 images in seven classes, and four images distributed in two classes were misclassified by Net-II, such classification ability is acceptable in engineering and manufacturing practice. Meanwhile, it is also clarified by such data that Net-II performs better on validation set than Net-I.

In summary, four CNN models showed undoubted learning ability on training and validation set and Net-IV had the best performance during training and validation.

### 5.3. Metrics and Evaluations of Testing Set

Generalization ability of CNN models was evaluated and compared using testing set composed of 67 images.

#### 5.3.1. Evaluations and Comparisons of Net-I and Net-II

Net-I and Net-II are evaluated using $F^{1}$ value which is commonly applied to multiclassification tasks. $F^{1}$ is defined based on two parameters: *precision* and *recall*, which are defined as follows:

(5) $$Precision_{h}=\frac{1}{n}\overset{n}{\underset{j\;=\;1}{\sum }}\frac{\left|\left\{x^{\left(i\right)}|y_{j}\in Y^{\left(i\right)}\wedge y_{j}\in h\left(x^{\left(i\right)}\right)\right\}\right|}{\left|\left\{x^{\left(i\right)}|y_{j}\in h\left(x^{\left(i\right)}\right)\right\}\right|},$$

(6) $$Recall_{h}=\frac{1}{n}\overset{n}{\underset{j\;=\;1}{\sum }}\frac{\left|\left\{x^{\left(i\right)}|y_{j}\in Y^{\left(i\right)}\wedge y_{j}\in h\left(x^{\left(i\right)}\right)\right\}\right|}{\left|\left\{x^{\left(i\right)}|y_{j}\in Y^{\left(i\right)}\right\}\right|},$$

for 1≤i≤m. Where, *n* is the number of classes, *m* is the number of examples, $x^{\left(i\right)}$ denotes the feature vector extracted from the *i*-th example, $Y^{\left(i\right)}$ denotes the set of the ground truth label associated with $x^{\left(i\right)}$, $y_{j}$ is the *j*-th class label, |⋅| is interpreted as the operation that returns the cardinality, h(x) is the classifier that returns the set of proper label of x. *precision* and *recall* are usually comprehensively considered and jointly used in practice. $F^{\beta }$ is an integrated version of *precision* and *recall* with the balancing factor β>0. $F^{\beta }$ is defined as Equation (7):

(7) $$F^{\beta }=\frac{\left(1+\beta^{2}\right)\cdot Precision_{h}\cdot Recall_{h}}{\beta^{2}\cdot Precision_{h}+Recall_{h}},$$

when β=1, $F^{\beta }$ returns the harmonic mean of *precision* and *recall* recognized as $F^{1}$:

(8) $$F^{1}=2\times \frac{Precision_{h}\cdot Recall_{h}}{Precision_{h}+Recall_{h}}.$$

The values of *precision*, *recall* and $F^{1}$ of Net-I and Net-II are listed in Table 12.

Apparently, precision, recall and $F^{1}$ of Net-II are all equal to 1, the reason is this network correctly classified all testing images. Such three indices of Net-I are a little lower than that of Net-II.

Specifically, top-3 confidence scores and corresponding classes of testing images which were misclassified and images which network models returned low confidence score of ground truth label being the proper label are illustrated in Table 13 and Table 14 (corresponding labels of the specific confidence score are listed in bracket).

It is evidently illustrated by above two tables that seven out of 67 testing images were misclassified by Net-I and there exists three images that Net-I returned low confidence score (lower than 60%) of ground truth label being the proper label; all the testing images were correctly classified by Net-II and there only exists one image that gained the confidence score lower than 60% of ground truth label being the proper label. The ground truth labels of aforementioned two kinds of images are highly consistent with that of misclassified validation images.

To summarize, compared with Net-I, generalization performance of Net-II is much better. Overfitting phenomenon appeared on CNN model which applied AlexNet structure, this is also in line with the consideration we discussed in Section 4.3 when establishing network models.

#### 5.3.2. Evaluations and Comparisons of Net-III and Net-IV

Multilabel classification tasks usually cannot directly use existing evaluation metrics for multiclassification problems, here we introduce mean interpolated average precision (*MiAP*) which is commonly applied in information retrieval community. The definition of *MiAP* is also based on *precision* and *recall*, but it is worth noting that such two indices are defined differently here from those in Section 5.3.1. We redefine *precision* and *recall* as Equations (9) and (10):

(9) $$Precision_{f}=\frac{\left|\left\{x^{\left(i\right)}|rank_{f}\left(x^{\left(i\right)},y_{j}^{\left(i\right)}\right)\leq rank_{f}\left(x^{\left(k\right)},y_{j}^{\left(k\right)}\right)\right\}\cap^{\;}\left\{x^{\left(i^{\prime }\right)}|y_{j}\in Y^{\left(i^{\prime }\right)},1\leq i^{\prime }\leq m\right\}\right|}{\left|\left\{x^{\left(i\right)}|rank_{f}\left(x^{\left(i\right)},y_{j}^{\left(i\right)}\right)\leq rank_{f}\left(x^{\left(k\right)},y_{j}^{\left(k\right)}\right)\right\}\right|},$$

(10) $$Recall_{f}=\frac{\left|\left\{x^{\left(i\right)}|rank_{f}\left(x^{\left(i\right)},y_{j}^{\left(i\right)}\right)\leq rank_{f}\left(x^{\left(k\right)},y_{j}^{\left(k\right)}\right)\right\}\cap^{\;}\left\{x^{\left(i^{\prime }\right)}|y_{j}\in Y^{\left(i^{\prime }\right)},1\leq i^{\prime }\leq m\right\}\right|}{\left|\left\{x^{\left(i^{\prime }\right)}|y_{j}\in Y^{\left(i^{\prime }\right)},1\leq i^{\prime }\leq m\right\}\right|},$$

for i=1,2,…,k  and k=1,2,…,m. Where, *m* is the sample size, $y_{j}^{\left(i\right)}$ denotes the label with one-hot encoding label with one-hot encoding for $x^{\left(i\right)}$ of the *j*-th class, $y_{j}^{\left(i\right)}\in \left\{0,1\right\}$, $f\left(x^{\left(i\right)},y_{j}^{\left(i\right)}\right)$ returns the confidence score of $y_{j}^{\left(i\right)}$ being a proper label of $x^{\left(i\right)}$, $rank_{f}\left(x^{\left(i\right)},y_{j}^{\left(i\right)}\right)$ returns the rank of confidence score deduced from $f\left(x^{\left(i\right)},y_{j}^{\left(i\right)}\right)$ based on descending order. For the *j*-th class label $y_{j}$, $\left\{x^{\left(i^{\prime }\right)}|y_{j}\in Y^{\left(i^{\prime }\right)},1\leq i^{\prime }\leq m\right\}$ is actually the set of examples of which class *j* is one of their labels. For the *j*-th class, $\left\{x^{\left(i\right)}|rank_{f}\left(x^{\left(i\right)},y_{j}^{\left(i\right)}\right)\leq rank_{f}\left(x^{\left(k\right)},y_{j}^{\left(k\right)}\right)\right\}$ include the examples with top-*k* confidence scores. Rather than recognizing examples with the highest confidence score or with the confidence score higher than a specific fixed threshold as positives, this operation in fact specifies changing threshold of confidence score as *k* varies from 1 to *m*, examples with confidence scores higher than that of the *k*–th example is recognized as positives after the descending ordering of confidence scores.

The number of the *recall* values we obtained from *j*-th class is $\left|\left\{x^{\left(i^{\prime }\right)}|y_{j}\in Y^{\left(i^{\prime }\right)},1\leq i^{\prime }\leq m\right\}\right|$, various *precision* values correspond to one *recall* level. Interpolation method is used to calculate average *precision* to simplify calculation and comprehensively consider the performance of models reflected by *precision* and *recall*. The interpolated precision $P_{interp}$ at each certain *recall* level R is defined as the highest *precision* value P retrieved for any *recall* level $R^{\prime }\geq R\;$:

(11) $$P_{interp}\left(R\right)=\underset{R^{\prime }\geq R}{\max}P\left(R^{\prime }\right),$$

for $R=\frac{1}{\left|\left\{x^{\left(i^{\prime }\right)}|y_{j}\in Y^{\left(i^{\prime }\right)},1\leq i^{\prime }\leq m\right\}\right|},\frac{2}{\left|\left\{x^{\left(i^{\prime }\right)}|y_{j}\in Y^{\left(i^{\prime }\right)},1\leq i^{\prime }\leq m\right\}\right|},\ldots ,1.$

Therefore, for the *j*-th class, interpolated average precision (*iAP*) could be calculated as Equation (12):

(12) $$iAP=\frac{1}{\left|\left\{x^{\left(i^{\prime }\right)}|y_{j}\in Y^{\left(i^{\prime }\right)},1\leq i^{\prime }\leq m\right\}\right|}\overset{\left|\left\{x^{\left(i^{\prime }\right)}|y_{j}\in Y^{\left(i^{\prime }\right)},1\leq i^{\prime }\leq m\right\}\right|}{\underset{i\;=\;1}{\sum }}P_{interp}\left(R\right).$$

*MiAP* of CNN models can be then calculated from the mean values of all classes:

(13) $$MiAP=\frac{1}{n}\overset{n}{\underset{j\;=\;1}{\sum }}iAP.$$

**Figure 6:**
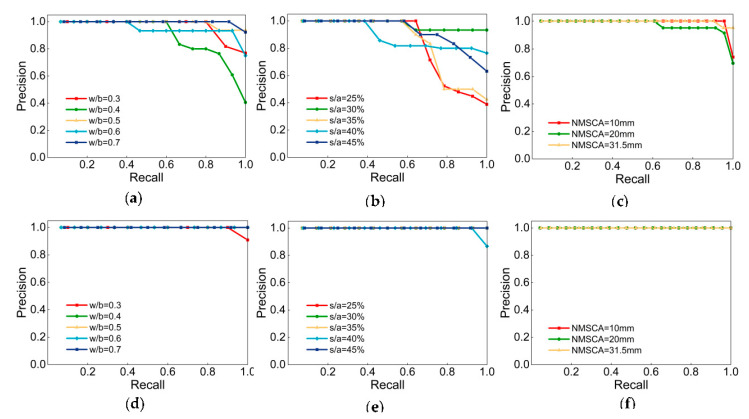
P–R curves of: (**a**) classes in w/b label of Net-III; (**b**) classes in s/a label of Net-III; (**c**) classes in NMSCA label of Net-III; (**d**) classes in w/b label of Net-IV; (**e**) classes in s/a label of Net-IV; (**f**) classes in NMSCA label of Net-IV.

Figure 6 illustrates the P–R curves of Net-III and Net-IV. MiAP and iAP of each class are manifested in Table 15. Definitely, both two CNNs achieved high MiAP and iAP, classification task was accomplished. Compare from the perspective of labels, the label NMSCA had higher P–R curves, indicate that networks had the best generalization ability with respect to NMSCA and two networks were of slightly inferior performance when identify w/b and s/a. With respect to comparation CNN models, MiAP and iAP of Net-III is lower than that of Net-IV, generalization ability of networks with AlexNet structure is relatively poor and there may be an overfitting phenomenon which is also consistent with the considerations discussed in Section 4.3.

*MiAP* is actually a label-based ranking metric only focusing on classification performance of classes of a model. However, in manufacturing and engineering practice, for a given sample, more attention is usually concentrated on error between the result identified by models and the actual situation. Although the essence of two CNNs are classifiers which output the confidence score of a specific class being proper label of a given image, three labels for classification in the present study are quantifiable indices. Hence, “identified values” of w/b, s/a and NMSCA of each testing image could be calculated by weighted average calculation using confidence scores and corresponding numeric values of classes in one mix proportion label. Correspondingly, “actual values” refer to the real w/b, s/a and NMSCA values reflected by ground truth label. Identified values could be compared with actual values by various indices to evaluate their errors. In this study, mean absolute percentage error (*MAPE*), absolute fraction of variance ($R^{2}$) were applied for error evaluation. For each class, *MAPE* and $R^{2}$ are defined as Equations (14) and (15):

(14) $$MAPE=\frac{1}{m}\overset{m}{\underset{i\;=\;1}{\sum }}\frac{a_{i}-p_{i}}{a_{i}}\times 100\%,$$

(15) $$R^{2}=1-\frac{\sum_{i\;=\;1}^{m}{\left(p_{i}-a_{i}\right)}^{2}}{\sum_{i\;=\;1}^{m}a_{i}{}^{2}},$$

where, $a_{i}$ and $p_{i}$ denote the actual value and identified values of the *i*-th example, *m* is the sample size.

**Figure 7:**
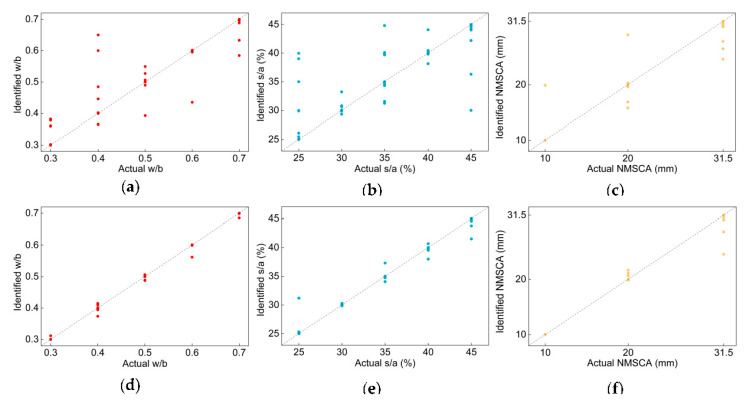
Identified: (**a**) w/b, (**b**) s/a, (**c**) NMSCA values of each testing image calculated with the outputs of Net-III and identified (**d**) w/b, (**e**) s/a, (**f**) NMSCA values of each testing image calculated with the outputs of Net-IV.

Actual and detected values of each testing image are illustrated in Figure 7. Undeniably, most images are on or close to the diagonal of the diagram. *MAPE* and $R^{2}$ of each label and average *MAPE* and $R^{2}$ of two networks are listed in Table 16. Apparently, *MAPE* of each label and two CNNs are small, even close to 0, $R^{2}$ values are close to 1. In contrast to *MiAP*, compared with w/b and s/a, the NMSCA label did not have the best performance when evaluating with *MAPE* and $R^{2}$, this is because the numeric difference of three classes in NMSCA label is larger than that in w/b or s/a label. When it comes to the performance of networks, comparison results are in line with that of *MiAP*, that is, Net-IV had better generalization ability.

**Figure 8:**
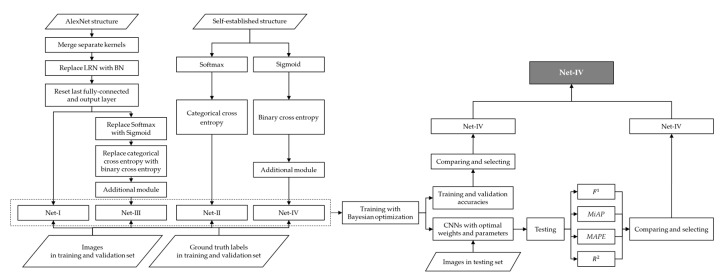
Flow chart of research methodology and results evaluation.

Aforementioned research methodology and results evaluations and comparations are visualized in the form of flow chart in Figure 8.

### 5.4. Comparative Study

Above arguments indicate that all the four CNN models have learned the features of mix proportion in fresh concrete images, presenting outstanding learning and generalization ability. Moreover, identification time of testing images is within 1 s, realizing real-time monitoring of mix proportion. Demonstrated by comprehensive comparisons, Net-IV was chosen as the representative achievement of the present study since it had undeniable the best performance. Net-IV was further named as ConcMPNet.

ConcMPNet was compared with methods proposed by Ref. [17,18,19,20] and existing testing method, as manifested in Table 17. Visibly, ConcMPNet requires the simplest approach. Real-time monitoring could be realized, green manufacturing could be implemented, and resource wasting problem was addressed only by ConcMPNet. Prominent merit and effectiveness of presented multilabel image classification and CNN-based monitoring method are highlighted by comparative studies.

### 5.5. Establishing of Mix Proportion Monitoring and Integrated Intelligent Sensing System

**Figure 9:**
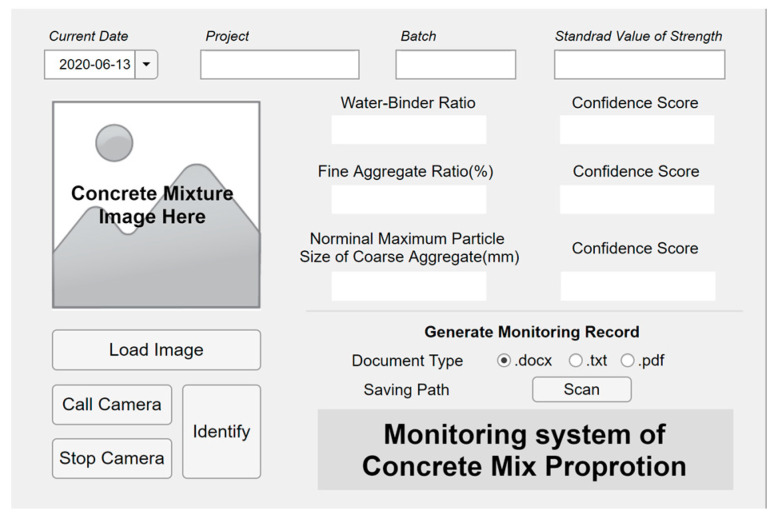
User interface of concrete mix proportion monitoring system.

ConcMPNet was embedded into executable application, visual interactive interface was designed and laid out, then the executable application was packaged and equipped with certain hardware facilities such as high-definition camera and deployed to terminals to establish fresh concrete mix proportion monitoring system, which could also be recognized as an intelligent sensor. The monitoring system has two parallel operation processes for sensing: taking real-time pictures and loading stored images in terminals. The user interface of established monitoring system is shown in Figure 9.

Such a system cooperated with existing mechanical and weighing sensors to establishing integrated intelligent sensing system. When production equipment fails to produce concrete mixture with proper calculated mix proportion, our integrated intelligent sensing system could send warning messages to fixed and mobile terminals. Real-time and full-scale mix proportion monitoring and inaccurate mix proportion sensing and warning during manufacturing could be achieved.

**Figure 10:**
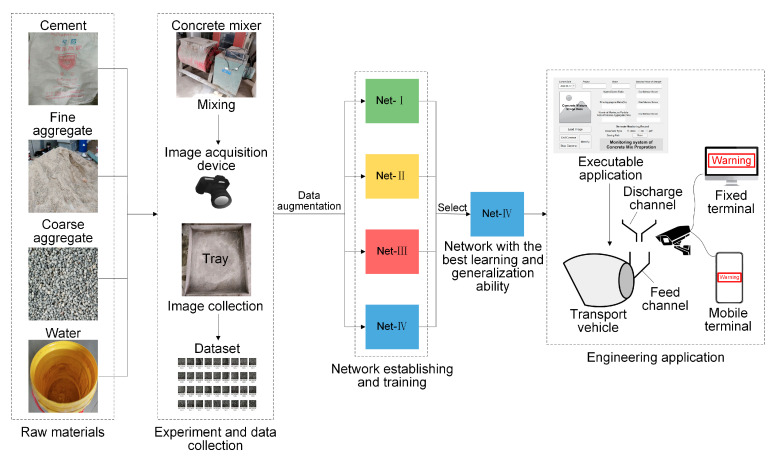
Working flow and research process of the present study.

To summarize, according to aforementioned series processes of CNN model establishing, improving, training, testing, selection, as well as the establishing of integrated intelligent sensing system, working flow and research process of the present study are illustrated in Figure 10.

## 6. Conclusions

In the present study, a novel deep-learning-based method was presented for mix proportion real-time and full-scale monitoring of fresh concrete. As a typical data-driven, learning-based approach, the key insight of this method lies in feeding elaborately planned, uniformly distributed, widely covered and high-quality data to CNN models which are enabled to extract implicit features and generalizing such features to newly fed data for identification. Accounting for engineering and manufacturing practice, w/b, s/a and NMSCA were selected as variables of mix proportion, also the labels of concrete mixture images and the target objects of classification and mix proportion monitoring. A total of 67 experiments were conducted, and the same number sets of images were collected which were annotated with different combinations of classes in above three mix proportion labels. Four CNN models, based on problem transformation and algorithm adaption, respectively, were established, improved, trained and tested. Training and validation accuracies of four networks are all above 99%. As for testing set, $F^{1}$ value of Net-I is above 0.85, and Net-II even reaches 1; *MiAP* of Net-III and Net-IV are all above 0.9, *MAPE*s are small enough and $R^{2}$ values are close to 1. All the four networks showed outstanding learning and generalization ability. Net-IV was chosen as the representative achievement and named as ConcMPNet after comparison. ConcMPNet was embedded into executable application and equipped with hardware facilities to establish fresh concrete mix proportion monitoring system. Such system was deployed to terminals and cooperated with existing mechanical and weighing sensors to establish integrated intelligent sensing system, real-time and full-scale mix proportion monitoring and inaccurate mix proportion sensing and warning during manufacturing could be achieved.

The contributions of this research lie in:

- The improved CNN model was embedded in executable application for fresh concrete mix proportion monitoring, overcoming the defects of real-time and full-scale monitoring that have not been, to date, addressed by existing testing method and other studies;
- The presented CNN model could likely be scaled up for other concrete mixture identification tasks. Our well-trained CNN model could be applied for transfer learning which received much attention recent year because it effectively reduces computational costs. It is crucial for the success of transfer learning that the dataset of target task is similar with original training dataset, but existing successful CNNs did not apply professional concrete mixture dataset for training. Therefore, transfer learning using our CNN model provides a potential way for future similar concrete identification tasks. For a specific kind of concrete mixture such as recycled concrete or environmentally friendly concrete, monitoring could be implemented by simply feeding collected dataset to our CNN models and carrying out transfer learning;
- As a multi-disciplinary research, this study introduced a state-of-the-art method to ancient and traditional engineering manufacturing community and widened application fields of intelligent methods and deep learning techniques. The present study provides a solid foundation for future works in both disciplines.

The prominent merit of presented method lies in that it can realize real-time monitoring of fresh concrete mix proportion only by taking pictures which could not be achieved by previous studies and existing methods. However, the proposed multilabel-image-classification-based method does not intend to be a cure-all. The convenience of monitoring is bought at the expense of huge number of previous experimentations and onerous work of data collection. This is also the shortcoming of all data-driven methods. More precise identification requires more data and larger datasets.

Future works could be built on the united application of CNN and other intelligent algorithms, such as ANN, to realize concrete property especially mechanical property prediction with the route of “image–mix proportion–property” and moreover, it is promising to cooperate the proposed system with series of other approaches, such as chemical composition sensors, to promote the establishing of a more intelligent and precise sensing system for concrete early properties monitoring stepping forward.

## Figures and Tables

**Table 1:** Classes of mix proportion labels.

	Classes of Each Label						
**w/b**	**0.3**	**0.4**		**0.5**	0.6		0.7
**s/a (%)**	25	30		35	40		45
**NMSCA (mm)**	10		20			31.5	

**Table 2:** Eight extreme mix proportions excluded from experiments.

w/b	s/a (%)	NMSCA (mm)	w/b	s/a (%)	NMSCA (mm)
0.3	40	10	0.3	45	31.5
0.3	40	20	0.7	25	31.5
0.3	45	10	0.7	30	31.5
0.3	45	20	0.7	35	31.5

**Table 3:** Physical and mechanical properties of cement.

Property		Actual Value	Accepted Value
Grade		P.O. 42.5	
Date of production		May, 2019	
Fineness (in terms of specific surface area, m^2^/kg)		360	≥300
Initial setting time (min)		160	≥45
Final setting time (min)		260	≤600
Compressive strength (MPa)	3 d	19.9	≥17
	28 d	48	≥42.5
Flexure strength (MPa)	3 d	4.2	≥3.5
	28 d	9.9	≥6.5

**Table 4:** Physical properties of aggregates.

Property		Actual Value	Accepted Value
**Type**	Fine aggregate	Medium sand	
	Coarse aggregate	Crushed stone	
Particle size (mm)	Fine aggregate	0–4.75	
	Coarse aggregate	5–31.5	
Fineness module	Fine aggregate	2.6	[2.3,3.0]
	Coarse aggregate	–	–
Apparent density (kg/m^3^)	Fine aggregate	2650	≥2500
	Coarse aggregate	2650	≥2600
Loose bulk voidage (%)	Fine aggregate	41.5	≤44
	Coarse aggregate	45.3	≤47

**Table 5:** Size grading of coarse aggregate.

Nominal Particle Size of Coarse Aggregate (mm)	Proportion of Each Size Grading (%)		
	5–10	10–20	20–31.5
5–10	100	–	–
5–20	50	50	–
5–31.5	30	50	20

**Table 6:** Detailed structure of Net-II.

Layer	Input Size	Kernel Size	Stride	Number of Filters	Processing
Conv1	256 × 256 × 3	11 × 11	4	64	ReLU, BN
Max pooling1	64 × 64 × 64	3 × 3	2	–	25% dropout
Conv2	31 × 31 × 64	5 × 5	1	128	ReLU, BN
Conv3	31 × 31 × 128	5 × 5	1	128	ReLU, BN
Max pooling2	31 × 31 × 128	3 × 3	2	–	25% dropout
Conv4	15 × 15 × 128	3 × 3	1	256	ReLU, BN
Conv5	15 × 15 × 256	3 × 3	1	256	ReLU, BN
Max pooling3	15 × 15 × 256	3 × 3	2	–	25% dropout
Dense1	7 × 7 × 256	–	–	–	ReLU, BN, 50% dropout
Dense2	1 × 1 × 2048	–	–	–	Softmax

**Table 7:** Searching sets of hyperparameters to be optimized.

Hyperparameter	Set of Search Values
Initial learning rate	{0.01,0.001,0.0001}
Batch size	{30,40,50,60,70,80,90}
Epoch	{80,90,100,110,120,130}

**Table 8:** Fixed training parameters and their options.

Training Parameter	Option
Optimizer	Adam
Learning rate drop period	Last 10 epoch
Learning rate drop factor	0.1
Shuffle	Every epoch
L2 regularization	0.0001
Execution environment	CPU
Validation frequency	1

**Table 9:** Adjustments or improvements of four CNN models.

Item	Net-I	Net-II	Net-III	Net-IV
**Basic structure**	AlexNet	Self-established	AlexNet	Self-established
**Adjustments on basic structure**	Merge separate kernels; Replace LRN with BN; Reset last fully connected and output layer.	–	Merge separate kernels; Replace LRN with BN; Reset last fully connected and output layer.	–
**Activation function of output layer**	Softmax	Softmax	Sigmoid	Sigmoid
**Loss function**	Categorical cross entropy	Categorical cross entropy	Binary cross entropy	Binary cross entropy
**Additional module**	–	–	Select the class with highest confidence score in each label	Select the class with highest confidence score in each label
**Hyperparameter tuning**	Bayesian optimization	Bayesian optimization	Bayesian optimization	Bayesian optimization
**Essence**	Multiclassification model	Multiclassification model	Multilabel classification model	Multilabel classification model

**Table 10:** Optimal hyperparameters obtained by Bayesian optimization.

	Batch Size	Epoch	Initial Learning Rate
Net-I	50	110	0.0001
Net-II	70	90	0.001
Net-III	80	100	0.001
Net-IV	60	100	0.001

**Table 11:** Training and validation accuracies of four CNN models.

	Training Accuracy (%)	Validation Accuracy (%)
Net-I	99.31	99.01
Net-II	99.78	99.88
Net-III	99.92	100
Net-IV	99.94	100

**Table 12:** Precision, recall and $F^{1}$ of Net-I and Net-II.

	*Precision*	*Recall*	$F^{1}$
Net-I	0.8433	0.8955	0.8686
Net-II	1	1	1

**Table 13:** Testing images which were misclassified by Net-I and images which Net-I returned low confidence score of ground truth label being the proper label.

Ground Truth Label			Top-3 Confidence Score (%) and Corresponding Class		
w/b	s/a (%)	NMSCA (mm)			
0.3	35	20	76.78 (0.4_30_10)	11.85 (0.3_35_20)	8.92 (0.4_30_31.5)
0.4	25	10	48.56 (0.3_25_10)	42.90 (0.4_25_10)	7.60 (0.6_30_10)
0.4	30	31.5	86.61 (0.4_30_20)	13.39 (0.4_30_31.5)	–
0.5	30	20	87.34 (0.3_30_10)	12.63 (0.5_30_20)	0.01 (0.5_40_10)
0.5	35	10	70.58 (0.5_30_10)	28.30 (0.5_35_10)	1.12 (0.3_25_10)
0.5	40	20	74.93 (0.5_25_31.5)	22.89 (0.5_40_20)	1.09 (0.3_25_31.5)
0.6	35	20	94.27 (0.6_40_10)	5.68 (0.6_35_10)	0.02 (0.3_30_20)
0.3	30	20	53.31 (0.3_30_20)	27.09 (0.4_40_10)	18.39 (0.3_25_31.5)
0.6	40	20	37.61 (0.6_40_20)	34.83 (0.6_35_20)	12.57 (0.6_45_10)
0.7	45	10	57.67 (0.7_45_10)	16.77 (0.7_35_10)	7.28 (0.6_30_10)

**Table 14:** Testing images which Net-II returned low confidence score of ground truth label being the proper label.

Ground Truth Label			Top-3 Confidence Score (%) and Corresponding Class		
w/b	s/a (%)	NMSCA (mm)			
**0.3**	35	20	46.17 (0.3_35_20)	40.45 (0.6_35_20)	8.37 (0.4_30_31.5)

**Table 15:** MiAP and iAP of each class and label.

Label	Class	Net-III			Net-IV		
		iAP	iAP of labels	MiAP	iAP	iAP of labels	MiAP
w/b	0.3	0.959	0.954	0.942	0.991	0.998	0.999
	0.4	0.881			1.000		
	0.5	0.988			1.000		
	0.6	0.948			1.000		
	0.7	0.994			1.000		
s/a	25	0.825	0.886		1.000	0.998	
	30	0.971			1.000		
	35	0.833			1.000		
	40	0.883			0.990		
	45	0.917			1.000		
NMSCA	10	0.989	0.985		1.000	1.000	
	20	0.969			1.000		
	31.5	0.996			1.000		

**Table 16:** MAPE and $R^{2}$ of each label and two network models.

	MAPE (%)				$R^{2}$			
	w/b	s/a	NMSCA	Average	w/b	s/a	NMSCA	Average
Net-III	−2.037	−3.014	−0.710	−1.920	0.989	0.987	0.991	0.989
Net-IV	0.141	−0.204	0.287	0.075	1.000	0.999	0.998	0.999

**Table 17:** Comparative study of ConcMPNet, method proposed by references and existing testing method.

Method	Approach	Time Required	Evaluation ^2^	Concrete Waste
ConcMPNet	Taking photos	Real time	0.998	×
Ref. [17]	pH measurement by ANC	100 d	0.990	√
Ref. [18]	Microwave permittivity measurement	2 d	0.88	√
Ref. [19] ^1^	Acousto-ultrasonic	2–90 d	≥90%	√
Ref. [20] ^1^	Microwave near-field noninvasive testing	2–9 d	0.966	√
Existing method	Mechanical experiment in laboratory	≥28 d	≥95%	√

^1^ Corresponding method only applied to measuring w/b, here the results of w/b measurement are compared with other methods; ^2^ For ConcMPNet and references [17,18], R^2^ is compared as evaluation metric; for reference [19], percentage of successful classification is adopted and more details could be referred in this study; reference [20] used R for evaluation; for existing testing method in laboratory, assurance rate no less than 95% is required for concrete compressive strength.
